# Transcriptomic and Non-Targeted Metabolomic Analyses Reveal the Flavonoid Biosynthesis Pathway in *Auricularia cornea*

**DOI:** 10.3390/molecules27072334

**Published:** 2022-04-04

**Authors:** Li Meng, Shaoyan Zhang, Xiaoran Bai, Xiaobo Li, Qingji Wang, Li Wang, Wei Wang, Zhuang Li

**Affiliations:** 1 Shandong Provincial Key Laboratory of Agricultural Microbiology, College of Plant Protection, Shandong Agricultural University, Tai’an 271018, China; mengli@sdau.edu.cn (L.M.); zhangshaoyan1995@126.com (S.Z.); 18769835516@163.com (X.B.); ericwong@sdau.edu.cn (Q.W.); haoyou0121@163.com (L.W.); uniwangwei@sdau.edu.cn (W.W.); 2 Shandong Mushroom Industiral Technology Innonation Research Institute, Jining 272000, China; lixiaobo1927@163.com

**Keywords:** edible fungi, wood ear, phenylpropanoid biosynthesis, coumarin biosynthesis, isoflavonoid biosynthesis

## Abstract

Flavonoids, which are abundant in plants, are recognized for their antioxidant and anticancer roles in clinical applications. However, little is known about the molecular basis of flavonoid biosynthesis in fungi. In this study, we found that inclusion of leachate of Korshinsk peashrub (*Caragana korshinskii*) in the fermentation medium increased the total flavonoid content of the edible fungus *Auricularia cornea* by 23.6% relative to that grown in a control medium. Combined transcriptomic and non-targeted metabolomic analysis of the flavonoid biosynthesis pathway in *A. cornea* illustrated that there are important metabolites in the phenylpropanoid, coumarin and isoflavonoid biosynthesis pathways. In addition, we found that certain homologous genes encode phenylalanine ammonia lyase (PAL), polyphenol oxidase (PPO) and chalcone isomerase (CHI) in these biosynthesis pathways. These results, in this study, provide a new line for studying the regulation of flavonoid production in edible fungi.

## 1. Introduction

Flavonoids are important secondary metabolites which can prevent or even ameliorate chronic diseases or disease symptoms such as cancer [1], diabetes [2] and inflammation [3,4]. Dietary flavonoids are present in many commonly consumed fruits, vegetables, grains, herbs and beverages [5]. Recently, mushrooms and other edible fungi have gained importance as functional foods, thus, creating a bridge between medicine and food [6]. Mushrooms and other edible fungi are known to accumulate a variety of secondary metabolites with antioxidant activities, such as phenolic compounds and flavonoids [7].

*Auricularia cornea* Ehrenb., previously named *A. polytricha* (Mont.) Sacc [8], is widely cultivated in China [9]. *Auricularia* spp. has long been used as a dietary supplement and natural-medicinal mushroom. The medicinal properties of this genus are primarily associated with the polysaccharides and flavonoids in their fruiting bodies [10].

Flavonoids are compounds derived from precursors originating via the phenylpropanoid pathway. Briefly, coumaroyl-CoA is synthesized from the amino acid phenylalanine by three enzymatic steps initiated by phenylalanine ammonia-lyase (PAL) [11]. Flavonoid synthesis starts by the condensation of 4-coumaroyl-CoA with three molecules of malonyl-CoA, yielding naringenin chalcone. This reaction is catalyzed by chalcone synthase (CHS). The generated chalcone is subsequently isomerized by chalcone flavanone isomerase (CHI) to yield a flavanone. From these central intermediates, the pathway diverges into several side branches, each yielding a different class of flavonoid, isoflavone, coumarin, etc. [12].

Several studies showed that flavonoid production is regulated by flavonoid structural genes [13] and transcription factors [14], leading to differences in the yield and type of flavonoid [15]. However, these studies primarily focused on plants, and there are few reports concerning the influence on fungal genes involved in flavonoid accumulation. Korshinsk peashrub (*Caragana korshinskii* Kom.), a drought-tolerant, perennial mesquite shrub, is widely distributed in northeastern, northwestern and northern China [16,17]. Significantly, there are abundant phenolic compounds in *Caragana*. For this reason, it is an extreme cultivation material for edible mushrooms. Whether the mushrooms can make use of these compounds remains unknown. Therefore, we detected the whole metabolome of the mycelia in the liquid medium with leachate of *C. korshinskii*. The aims of our study were twofold; one objective was to investigate if leachate from *C. korshinskii* used as the fermentation medium could increase the total flavonoid content of *A. cornea*. The second objective was to elucidate the flavonoid biosynthesis pathway in *A. cornea*.

## 2. Results

### 2.1. Overview of Qualitative Metabolomics Analysis of A. cornea

**Figure 1:**
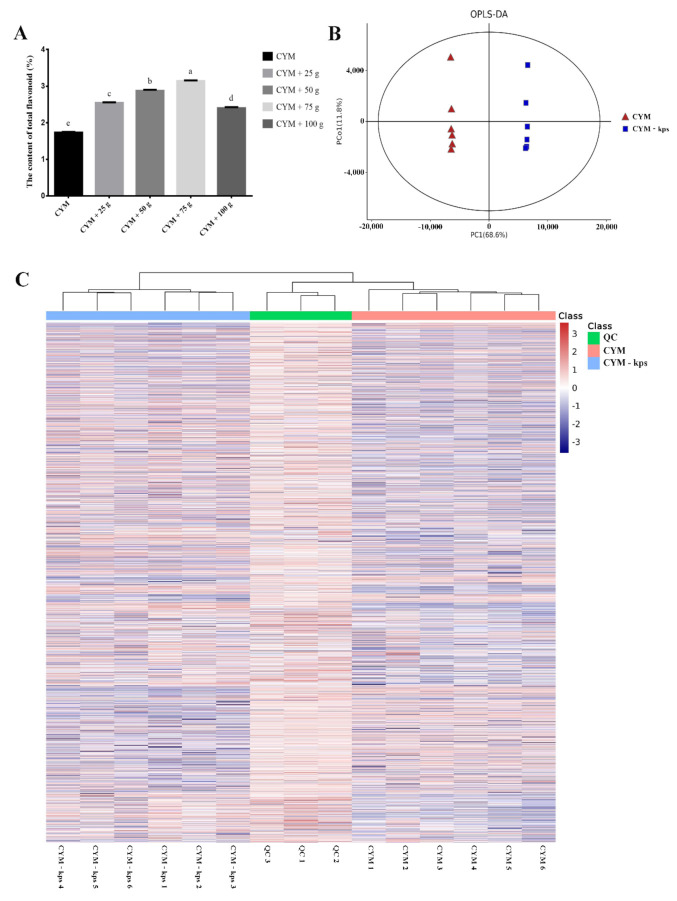
Qualitative metabolomics analysis of *A. cornea. (***A**) the content of total flavonoid. Means within a column carrying the same letter are not significantly different. Different superscript lowercase letters (a, b, c, d, and e) indicate significant differences (*p* < 0.05). CYM, complete yeast medium as control group; CYM + 25 g/L, CYM + 50 g/L, CYM + 75 g/L and CYM + 100 g/L, respectively, indicate CYM-kps medium with *C. korshinskii* leachate concentrations of 25 g/L, 50 g/L, 75 g/L and 100 g/L. (**B**) OPLS-DA analysis between different samples. (**C**) a hierarchical heatmap clustering analysis of the samples. QC, quality control sample; the QC sample was generated from a mixture of equivalent quantities of the 12 samples in this study. CYM, the metabolites of mycelia growth in the complete yeast medium. CYM-kps, the metabolites of mycelia growth in the CYM medium with *C. korshinskii* leachate concentration of 75 g/L.

There was a 23.6% increase (*p* < 0.05) in the total flavonoid content of *A. cornea* mycelia in the CYM-kps medium compared with that in the CYM medium. The concentration of total flavonoids was the highest, 3.14%, in mycelia cultured in CYM-kps medium with a leachate concentration of 75 g/L (Figure 1A). In order to fully elucidate the molecular basis of flavonoid biosynthesis in *A. cornea*, we cultured mycelia in CYM-kps medium (leachate concentration of 75 g/L) for all subsequent transcriptomic and non-targeted metabolomic analyses; mycelia cultured in CYM medium was used as the control.

The non-targeted metabolomic analysis was conducted to investigate, in more detail, the possible mechanism of flavonoid biosynthesis caused by media mixing leachate of Korshinsk peashrub. To compare the different metabolite compositions of *A. cornea* in different media, the mycelia of different media (CYM and CYM-kps) were subjected to an LC–MS analysis. The sample repetition correlation graph showed that the sample reproducibility was good (Figure 1B). A hierarchical heatmap clustering analysis of the samples showed that all the biological replicates were grouped together, indicating the good quality and high reliability of the metabolome data (Figure 1C). Metabolomics data were deposited to the EMBL-EBI MetaboLights database with the identifier MTBLS2337. The complete dataset can be accessed at https://www.ebi.ac.uk/metabolights/MTBLS2337 (accessed on 19 January 2021). The metabolome data can be used for subsequent analyses.

### 2.2. Overview of Qualitative Transcriptomic Analysis of A. cornea

**Figure 2:**
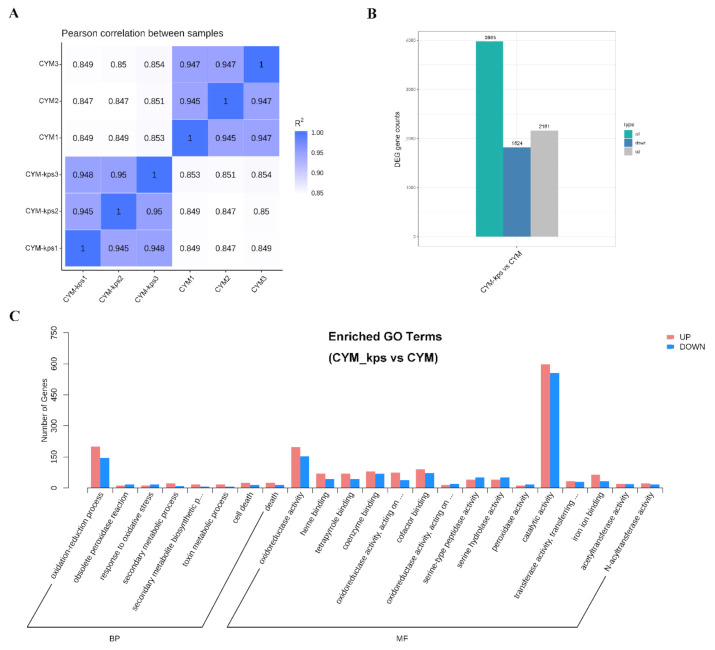
Qualitative transcriptomic analysis of *A. cornea.* (**A**) Pearson correlation between samples. (**B**) the differential expressed genes counts. (**C**) GO classification analysis of different samples. CYM, complete yeast medium as control group; CYM-kps, CYM medium with *C. korshinskii* leachate concentration of 75 g/L.

The results of Pearson correlation analysis between samples showed that the sample reproducibility was good (R^2^ > 0.8), and the transcriptomic data can be used for subsequent analyses (Figure 2A). Transcriptome data were deposited in the NCBI database with the identifier SAMN17169317-SAMN17169328. The complete dataset can be accessed at https://www.ncbi.nlm.nih.gov/sra/17169317-17169328 (accessed on 21 December 2020).

A total of 3985 different expression genes between CYM and CYM-kps were detected. There were 1824 and 2161 genes down-regulated and up-regulated in the different media, respectively (Figure 2B). All of differential expressed genes were analyzed by GO classification (Figure 2C). The results showed the differential expressed genes were mainly related to the oxidation–reduction process, oxidoreductase activity and catalytic activity, which increased the number of up-regulated genes more than down-regulated genes. In addition, most of the differential expressed genes involved in the secondary metabolic process were up-regulated. The results indicated that a lot of biological process were changed in the different media and suggested that the secondary metabolisms were rearranged.

### 2.3. The Flavonoid Biosynthesis Pathway in A. cornea

**Figure 3:**
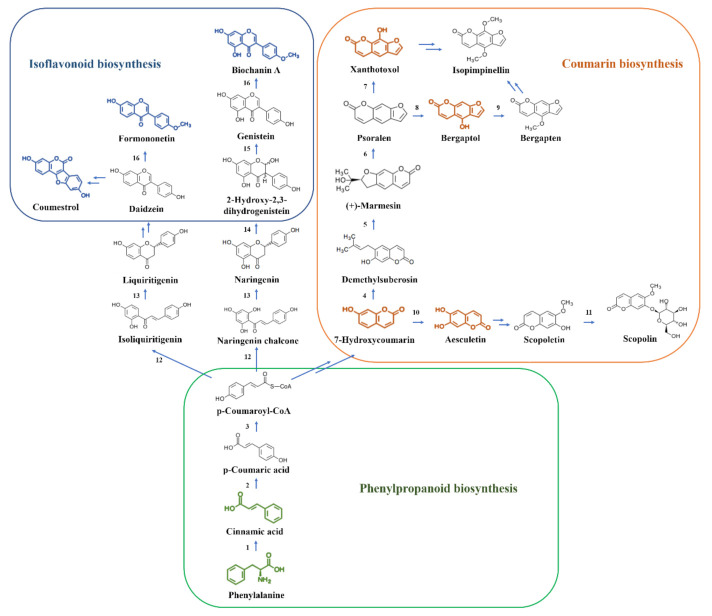
The flavonoid biosynthesis pathway in *A. cornea.*

Non-targeted metabolomic data analysis indicated nine metabolites in the flavonoid biosynthesis pathways, specifically in the phenylpropanoid biosynthesis, coumarin biosynthesis and isoflavonoid biosynthesis pathways. There were two metabolites, phenylalanine and cinnamic acid, in the phenylpropanoid biosynthesis pathway. There were four metabolites, 7-hydroxycoumarin, aesculetin, xanthotoxol and bergaptol, in the coumarin biosynthesis pathway. The other three metabolites were coumestrol, formonoetin and biochanin A in the isoflavonoid pathway. We mapped a flavonoid biosynthesis pathway in *A. cornea* (Figure 3).

**Figure 4:**
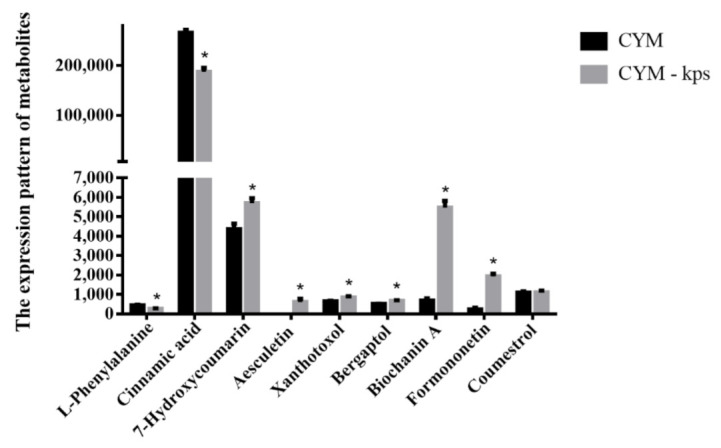
The expression pattern of metabolites identified in *A. cornea.*

Metabolomic analysis revealed that the expression patterns of the majority of the nine metabolites identified to be involved in the flavonoid biosynthesis pathway in *A. cornea* were significantly higher in mycelia cultured in the CYM-kps medium relative to the control (Figure 4, Supplementary Material S1–S2). The expression patterns of 7-hydroxycoumarin, xanthotoxol and bergaptol in the coumarin biosynthesis pathway were 1.31-fold, 1.30-fold and 1.31-fold higher, respectively. Interestingly, aesculetin was not detected in control cultures but was present at a level of 606.11 in mycelia cultured in CYM-kps medium. The expression of biochanin A and formononetin was 8.01-fold and 8.56-fold higher, respectively, in CYM-kps compared to the control. However, no significant difference was observed regarding coumestrol expression. Finally, phenylalanine and cinnamic acid, which are important precursors of some flavonoids, were significantly down-regulated in the CYM-kps medium. A possible explanation for this observation is that they are involved in the coumarin and isoflavonoid biosynthesis pathways.

The aforementioned nine metabolites are depicted in different colors in Figure 3. Blue depicts the isoflavonoid biosynthesis pathway, orange indicates the coumarin biosynthesis pathway and green indicates the phenylpropanoid biosynthesis pathway. The numbers indicate enzymatic activity, and double arrows indicate multistep reactions. 1. Phenylalanine ammonia lyase (PAL); 2. Cinnamate 4-hydroxylase (C4H); 3. 4-coumaric acid coenzyme A ligase (4CL); 4. Umbelliferone 6-dimethylallyltransferase (U6DT); 5. Marmesin synthase (MS); 6. Psoralen synthase (PS); 7. Psoralen 8-hydroxylase (P8H); 8. Psoralen 5-hydroxylase (P5H); 9. Bergaptol 5-*O*-methyltransferase (B5OMT); 10. Polyphenol oxidase (PPO); 11. Scopoletin glucosyltransferase (SGT); 12. Chalcone synthase (CHS); 13. Chalcone isomerase (CHI); 14. 2-hydroxyisoflavanone synthase (IFS); 15. 2-hydroxyisoflavanone dehydratase (HIDH); 16. Isoflavone 4-*O*-methyltransferase (HI4OMT).

CYM, complete yeast medium as the control group; CYM-kps, CYM medium with leachate of Korshinsk peashrub (*Caragana korshinskii*) at a concentration of 75 g/L; * indicates statistical significance (*p* < 0.05) compared to the control.

### 2.4. The Expression Levels of the Genes Encoding Key Enzymes Involved in the Flavonoid Biosynthesis Pathway

**Figure 5:**
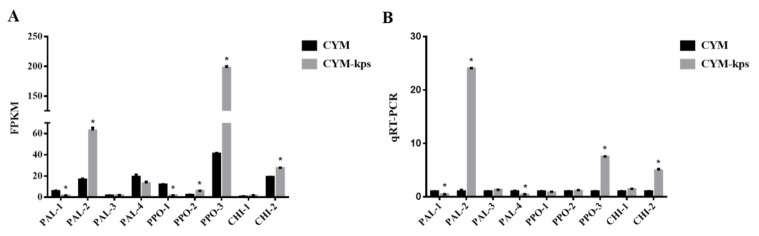
Transcript levels of the homologous genes encoding key enzymes involved in the flavonoid biosynthesis pathway in *A. cornea. (***A**) transcriptome data (FPKM), (**B**) relative gene expression levels (qRT-PCR). Error bars indicate the standard deviations across three independent samples; FPKM, expected number of fragments per kilobase of transcript sequence per million base pairs sequenced; CYM, complete yeast medium as the control group; CYM-kps, CYM medium with leachate of Korshinsk peashrub (*Caragana korshinskii*) at a concentration of 75 g/L; qRT-PCR, quantitative real-time polymerase chain reaction; * indicates statistical significance (*p* < 0.05) compared to the control.

There were four homologous genes, *PAL-1*, *PAL-2*, *PAL-3* and *PAL-4* (GenBank accession numbers: MW652285-MW652288), encoding phenylalanine ammonia lyase (PAL), three homologous genes, *PPO-1*, *PPO-2* and *PPO-3* (GenBank accession numbers: MW652289-MW652291), encoding polyphenol oxidase (PPO) and two homologous genes, *CHI-1* and *CHI-2* (GenBank accession numbers: MW922315, MW922316), encoding chalcone isomerase (CHI), identified by transcriptome analysis in *A. cornea*. The expression levels of *PAL-2*, *PPO-2*, *PPO-3* and *CHI-2* were significantly up-regulated (*p* < 0.05) in mycelia cultured in CYM-kps medium relative to the control (Figure 5A). The expression levels (FPKM) of *PAL-2*, *PPO-2*, *PPO-3* and *CHI-2* were 3.79-fold, 2.74-fold, 4.81-fold and 1.44-fold higher, respectively, in CYM-kps medium. On the other hand, the expression levels of *PAL-1*, *PAL-4* and *PPO-1* were down-regulated. Lastly, the expression levels (FPKM) of *PAL-3* and *CHI-1* were 1.22-fold and 2.63-fold higher, respectively, in CYM-kps medium, but these differences were not significant (*p > 0.05*). In addition to transcriptome analysis, we used qRT-PCR to compare these genes’ expression levels in between CYM-kps medium and the control (Figure 5B). The results of qRT-PCR analyses were consistent with the gene expression transcription analyses.

## 3. Discussion

The presence of flavonoids in edible fungi was once suspected in the past [18,19]. However, some reports found that flavonoids are widespread in edible fungi, for instance, *Fistulina hepatica* [20], *Hericium corallloides* [21], *Pleurotus florida* [22] and *Sanghuangporus vaninii* [23]. The doubt is due to lack of direct analytical evidence of the presence of flavonoids in edible fungi.

In fact, there are flavonoids in the edible fungi. Firstly, some studies proved the existence of a flavonoid biosynthesis pathway in edible fungi [24]. Several studies reported the total flavonoid content in edible fungi such as *Lentinus edodes* [25], *Volvariella volvacea* [26], *Tricholoma* spp. [27], *Pleurotus ostreatus* and *P. citrinopileatus* [28]. Secondly, a genome-wide survey across the fungal kingdom revealed the presence of all the gene/protein sequences associated with flavonoid biosynthesis [24]. They included phenylalanine ammonia lyase [29], chalcone isomerase and flavonol synthase [30]. This indicates that fungi synthesize enzymes associated with flavonoid biosynthesis. Finally, the contents of specific flavonoids, such as quercetin [20] and catechin [31], were measured in edible fungi by high-performance liquid chromatography (HPLC). However, there are no standard assays to quantify flavonoids in edible fungi, and the molecular basis of their biosynthesis remains unclear. To elucidate this in *A. cornea*, we used metabolomic sequencing by LC–MS. In addition, we compared the expression patterns of genes in the flavonoid biosynthesis pathway between mycelia cultured in CYM medium and CYM-kps medium. Our results illustrated that there are important metabolites in the flavonoid biosynthesis pathway in *A. cornea*, with transcriptome analysis indicating the involvement of homologous genes encoding PAL, PPO and CHI.

There are more abundant phenolic compounds which were shown to act as excellent antioxidants in *Caragana* [32]. Interestingly, these phenolic compounds, including flavonoids, gallic acid, chlorogenic acid and rutin, also exist in edible fungus *Auricularia* spp.; the results suggest that a submerged culture of *Auricula* is an effective method to produce total phenolics [33,34]. Findings were similar in our study; the results of metabolomic data showed that various phenolic compounds existed in the mycelia of *A. cornea.* Furthermore, previous studies reported that edible fungi can synthesize flavonoids, and flavonoid contents can be enriched by fermentation [35] or nitric oxide [36]. Consistent with these studies, we found that the flavonoid content in mycelia of *A. cornea* was enriched by fermentation through the addition of leachate of *C. korshinskii*. Unfortunately, our data have not identified a specific reason for enhancing the content of flavonoids. We suspect that the different levels of precursors or intermediate stimuli caused this difference. However, the regulation mechanism of leachate is unknown and worthy of further study.

The flavonoid biosynthetic pathway was partially mapped in *S. vaninii* [30]. However, there have been no studies on the biosynthesis of coumarin and isoflavones in fungi. In this study, non-targeted metabolomic and transcriptomic data confirmed the presence of nine metabolites in the flavonoid biosynthesis pathway, relating to phenylpropanoid, coumarin and isoflavonoid biosynthesis. These results provide a preliminary basis for the study of secondary metabolic biosynthesis pathways in edible and/or medicinal fungi.

## 4. Materials and Methods

### 4.1. Strains and Culture Conditions

An *A. cornea* strain, AC5, was provided by the Collection Center of Mushroom at Jilin Agricultural University. It was grown at 26 °C on potato dextrose agar (PDA) medium for 7 d and then inoculated into 100 mL complete yeast medium (CYM) (1% maltose, 2% glucose, 0.2% yeast extract, 0.2% tryptone, 0.05% MgSO_4_·7H_2_O, 0.46% KH_2_PO_4_). It is widely known that *Caragana* is rich in phenolic compounds, and it is an extreme cultivation material for edible mushrooms. Whether the mushroom can make use of these compounds remains unknown. Therefore, we detected the whole metabolome of the mycelia in the CYM and CYM with leachate of *Caragana*. It is important to note, however, that there are several tannin substances contained in the leachate of Korshinsk peashrub (*Caragana korshinskii*) which are affected the fungal mycelial growth. Due to this, we performed a screening in four different leachate concentrations in our initial experiments. The mycelia were pre-cultured for 3 d in CYM medium. The branches of Korshinsk peashrub were collected from the Ningxia province in August. The leachate of Korshinsk peashrub was from branches extract. Briefly, the branches of Korshinsk peashrub were cut into small segments of about 2 cm. Distilled water (1L) was added to the different weights (25 g, 50 g, 75 g and 100 g) of Korshinsk peashrub and boiled for 15 min. After filtration, the solution was collected, and the volume was set to 1 L by adding distilled water. The leachate concentrations were 25 g/L, 50 g/L, 75 g/L and 100 g/L, respectively. Following extraction, the 100 mL CYM medium was mixed with 50 mL of leachate of Korshinsk peashrub at several different concentrations as CYM-kps medium, and CYM medium was used as a control (total volume was 150 mL). Mycelia cultures were shaken (160 rpm) at 26 °C for 6 d. The collected mycelia were frozen in liquid nitrogen for RNA extraction and were dried to a constant weight at 60 °C for flavonoid content determination.

### 4.2. Determination of Total Flavonoid Content

The total flavonoid content was measured using a modified method [37]. Briefly, 0.3 g of dried, ground mycelium was suspended in 10 mL of 100% ethyl alcohol. The mixture was agitated for 1 h using ultrasound. Three milliliters of the extracts was diluted with an additional 12 mL absolute ethyl alcohol. Subsequently, the mixture was sequentially mixed with 1 mL aluminum nitrate (100 g/L, aqueous) and 1mL potassium acetate (98 g/L, aqueous). Finally, the mixture was diluted with distilled water to a total volume of 50 mL and then allowed to rest for 1 h at room temperature. The absorbance at 420 nm was then measured spectrophotometrically. Absolute ethyl alcohol was used as the blank. A standard curve of rutin was graphed based on concentrations of 0.06, 0.13, 0.20, 0.27 and 0.33 mg/mL. The concentration of total flavonoids on a dry matter basis was calculated using the following formula:

X = m/(W × d × 1000) × 100%(1)

where ‘X’ is the total flavonoid content, ‘m’ is the quantity of rutin according to the standard curve (mg), ‘W’ is the quantity of the sample (g) and ‘d’ is the dilution rate.

### 4.3. Sample Preparation and LC–MS Analysis

Mycelia cultures were shaken (160 rpm) in CYM and CYM-kps media at 26 °C for 9 d (including 3 d pre-culture). Thirty milligrams of mycelial sample, collected by filtration, was extracted by adding 1 mL of a solution composed of methanol–water (7: 3, *v/v*). Subsequently, two 5 mm steel beads were added to the solution, placed in a fully automatic sample fast grinding machine, Wonbio-E (Shanghai, China), and processed for 2 min at 60 Hz. Ultrasonic extraction was performed in an ice-water bath for 30 min, after which, extracts were stored overnight at −20 °C. Extraction was centrifuged at 13,000 rpm for 10 min. The supernatants were collected, filtered through 0.22 µm polyvinylidene fluoride membrane and stored at −80 °C until subsequent LC–MS analysis. An internal standard of 2-chloro-L-phenylalanine (0.3 mg/mL), dissolved in methanol, was added to all sample extracts.

To identify mycelial metabolites, 12 samples from three independent biological replicates were randomly analyzed by LC–MS analysis. An ACQUITY UPLC I-Class system (Waters Corporation, Milford, MA, USA) coupled with VION IMS QTOF mass spectrometer (Waters Corporation, Milford, MA, USA) was used to analyze the metabolic profile in both ESI-positive and ESI-negative ion modes. Chromatographic separation of samples was performed using an ACQUITY UPLC HSS T3 column (2.1 mm × 100 mm, 1.8 μm). The column temperature was maintained at 45 °C. Mobile phase A was 0.1% formic acid in deionized water, and mobile phase B was acetonitrile containing 0.1% formic acid. The elution profile was set as follows: 0.01 min, 5% B; 2 min, 5% B; 4 min, 30% B; 8 min, 50% B; 10 min, 80% B; 14 min, 100% B; 15.1 min, 5% B; and 16 min, 5% B. The flow rate was 0.35 mL/min, and the injection volume was 2 μL. Instrument settings were as follows: ion source: ESI; capillary temperature: 320 °C; spray voltages: (+3.5, −3) kV; mass scan range: 125–1000; resolution (full scan): 70,000; resolution (HCD MS/MS scans): 17,500; sheath gas flow rate (Arb): 40 (positive ion) and 35 (negative ion); aux. gas flow rate (Arb): 10 (positive ion) and 8 (negative ion).

### 4.4. Metabolite Identification and Quantification

Raw data were collected using Software UNIFI 1.8.1. Baseline filtration, peak identification, peak alignment, peak filling, retention time (RT) and normalization of the raw data were performed by Progenesis QI v2.3 (Waters Corporation, Milford, MA, USA). Metabolite identifications based on exact mass-to-charge ratios (*m/z*), isotope distributions, fragmentation patterns and database hits (The Human Metabolome Database, Lipidmaps and METLIN) were performed. Additionally, the self-written R package and in-house, self-built secondary mass spectrometry database, which contained 550 metabolites, was applied in metabolite identification. Data processing parameters were applied as follows: precursor tolerance: 5 ppm; fragment tolerance: 10 ppm; and product ion threshold: 5%. Compounds with more than 50% missing values for each condition were removed, and the remaining missing values were replaced by half of the minimum value. Qualitative data were analyzed according to the score of qualitative results. Compounds with a score of more than 36 (a full score of 60) were accepted, whereas those with a score of less than 36 were deleted. The score consisted of 20 points for MS/MS matching, 20 points for MS/MS fragmentation matching and 20 points for isotopic distribution matching, with a maximum total score of 60 points. In addition, a mixture of 12 samples with equivalent quantities was used as quality control (QC) samples, and the QC samples were injected to monitor the stability of the analysis. Further, R^2^Y, Q^2^Y and 200-permutation tests were used to evaluate the quality of the orthogonal partial least squares discriminant analysis (OPLS-DA) mode. Metabolomics data were deposited to the EMBL-EBI MetaboLights database [38].

### 4.5. Transcriptome Sequencing Analysis

A total amount of 1.5 µg RNA per sample was used as the input for RNA sample preparation. Sequencing libraries were generated using NEBNext^®^ Ultra^™^ RNA Library Prep Kit for Illumina^®^ (NEB, Ipswich, MA, USA). The clustering of the index-coded samples was performed on a cBot Cluster Generation System using TruSeq PE Cluster Kit v3-cBot-HS (Illumina) according to the manufacturer’s instructions. After cluster generation, the library preparations were sequenced on an Illumina Hiseq platform and paired-end reads were generated.

Raw data (raw reads) in fastq format were first processed through in-house perl scripts. In this step, clean data (clean reads) were obtained by removing reads containing adapters, reads containing poly-N and low-quality reads from raw data. At the same time, the Q20, Q30, GC-content and sequence duplication level of the clean data were calculated. All of the downstream analyses were based on clean, high-quality data.

Gene function was annotated based on the following databases: Nr (NCBI non-redundant protein sequences); Nt (NCBI non-redundant nucleotide sequences); Pfam (Protein family); KOG/COG (Clusters of Orthologous Groups of Proteins); Swiss-Prot (A manually annotated and reviewed protein sequence database); KO (KEGG Ortholog database); and GO (Gene Ontology).

### 4.6. Gene Expression Analysis and Statistical Analysis

To evaluate the genes encoding key enzymes in the flavonoid synthesis pathway in *A. cornea*, total RNA was extracted from samples using the RNAiso Plus Kit (Takara, Kusatsu, Japan) according to our previous study [39]. First-strand cDNA was synthesized from total RNA using the HiScript III RT SuperMix for qPCR (+gDNA wiper) (TransGen Biotech, Beijing, China). We performed qRT-PCR using a LightCycler 96 SW 1.1 instrument with ChamQ Universal SYBR qPCR Master Mix (Vazyme, Nanjing, China) to analyze the transcription levels of genes based on the relative quantitation method. The gene expression level of polyubiquitin (*UBQ*) was found to be stable under experimental conditions and was accordingly used as the reference gene [40]. The primers for qRT-PCR analysis were designed using Primer5.0 software and are listed in Table 1. The cycling reaction conditions were as follows: 30 s at 95 °C, 40 cycles of 10 s at 95 °C and 30 s at 60 °C. The relative expression levels of specific genes were calculated according to the 2^−^^△△ct^ method [41]. Three biological replicates and three technical replicates were examined. The statistical significance of differences in results was tested with IBM SPSS Statistics 20.

## 5. Conclusions

The leachate of Korshinsk peashrub (*Caragana korshinskii*) in the fermentation medium increased the total flavonoid content of the edible fungus *Auricularia cornea*. Combined transcriptomic and non-targeted metabolomic analysis of the flavonoid biosynthesis pathway in *A. cornea* illustrated that there are important metabolites in the phenylpropanoid, coumarin and isoflavonoid biosynthesis pathways. In addition, we found that certain homologous genes encode PAL, PPO and CHI in these biosynthesis pathways. These results, in this study, provide a new line for studying the regulation of flavonoid production in edible fungi.

## Figures and Tables

**Table 1:** The primers for qRT-PCR analysis in this study.

Genes	Forward Primer Sequence (5′-3′)	Reverse Primer Sequence (5′-3′)
*UBQ*	CGGATCTAACAGCGTGGACTCTTC	CCTCCTGAGCGATTGGCACTTG
*PAL-1*	CGCCAATCAGGTCGCTAT	TTGCTGCTTCGTCGGC
*PAL-2*	GCAGGCTCCTTCCATCC	AGGCACCTCCTCATCGTC
*PAL-3*	AGTACGACGCACTGGGC	CGCATCTGCTCTATCACG
*PAL-4*	CGCAGATGGAGGACA	AGCGAGACGAACGAAGC
*PPO-1*	TCGTGGCTTCAGATTGG	GCGACGCTTGCTTT
*PPO-2*	AGACCGATCAACTCAGGTT	CAGGACGGAGCGATACTT
*PPO-3*	ACGCTTCTCAACACCCTC	TTCGTCGCCAAACACC
*CHI-1*	GAGTCTGCCTCCACCTACC	TTGATCTCGGCCAGCA
*CHI-2*	CAATGGCTTGGGCTCGTT	CGTGCCGCTTCAGATGGT

## Data Availability

The data presented in this study are available on request from the corresponding author.

## Supplementary Materials

The following are available online at https://www.mdpi.com/article/10.3390/molecules27072334/s1, Supplementary meterial S1: The metabolites data in this study; Supplementary meterial S2: The HPLC chromatograms of metabolises in this study.

## Sample Availability

Samples are not available.
