# Supplementary figures and images for: Transcriptomic and Non-Targeted Metabolomic Analyses Reveal the Flavonoid Biosynthesis Pathway in Auricularia cornea
Source: Molecules. 2022 Apr 4;27(7):2334. doi: 10.3390/molecules27072334 (PMC9000485; doi:10.3390/molecules27072334)

## Supplementary material S2 The HPLC chromatograms of metabolises in this study.

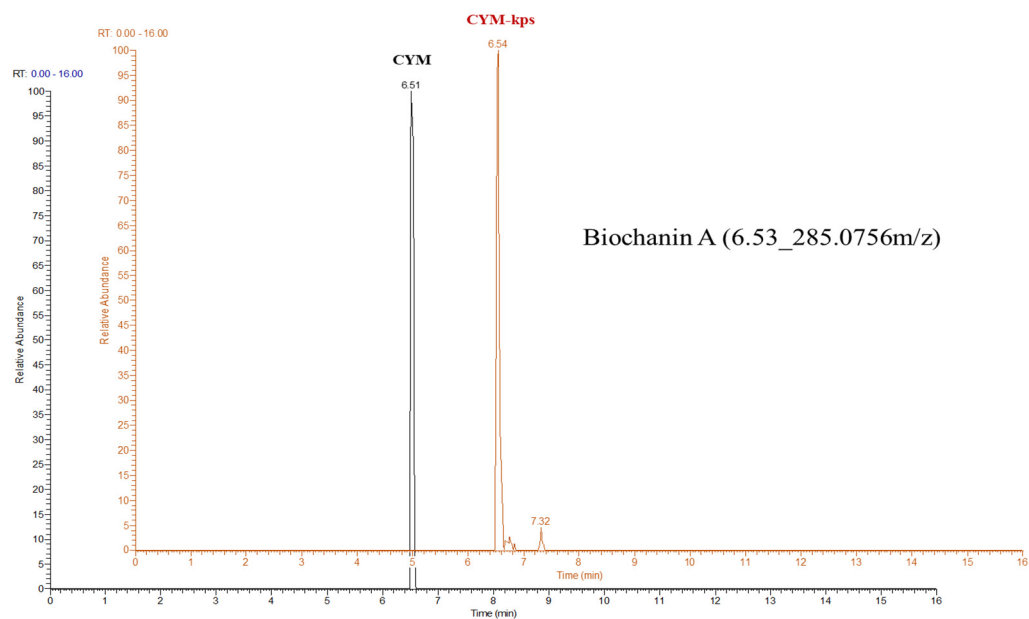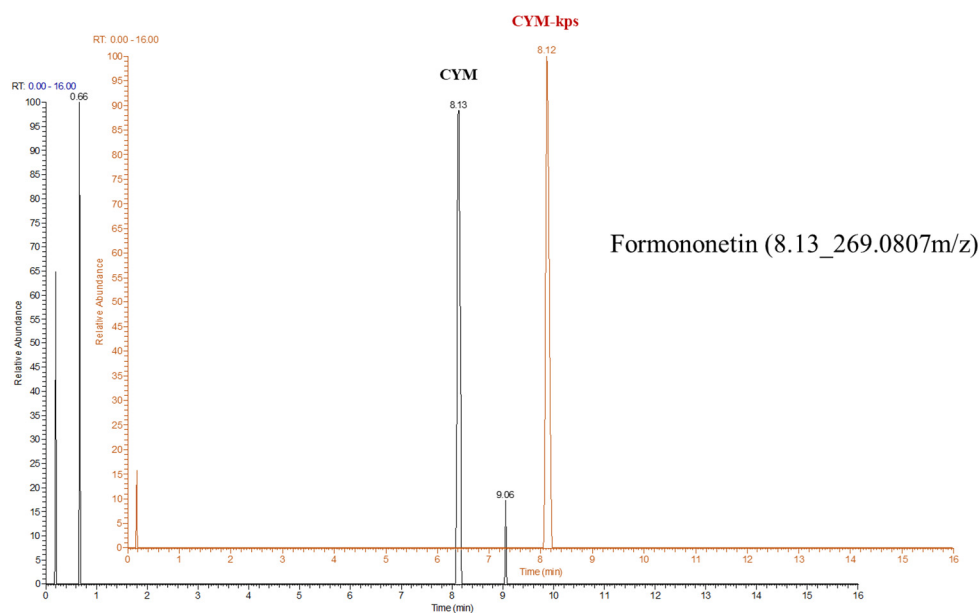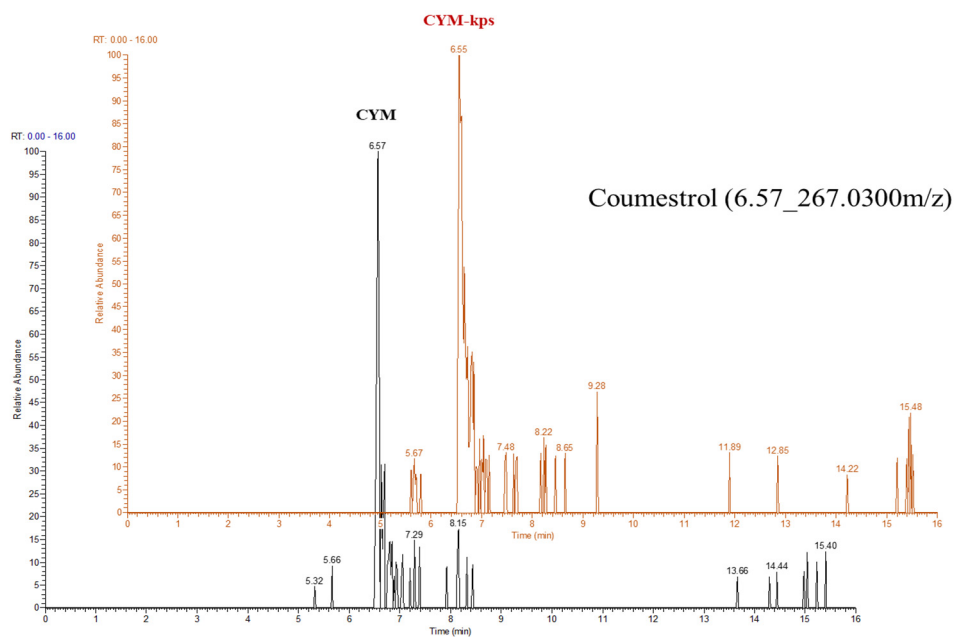

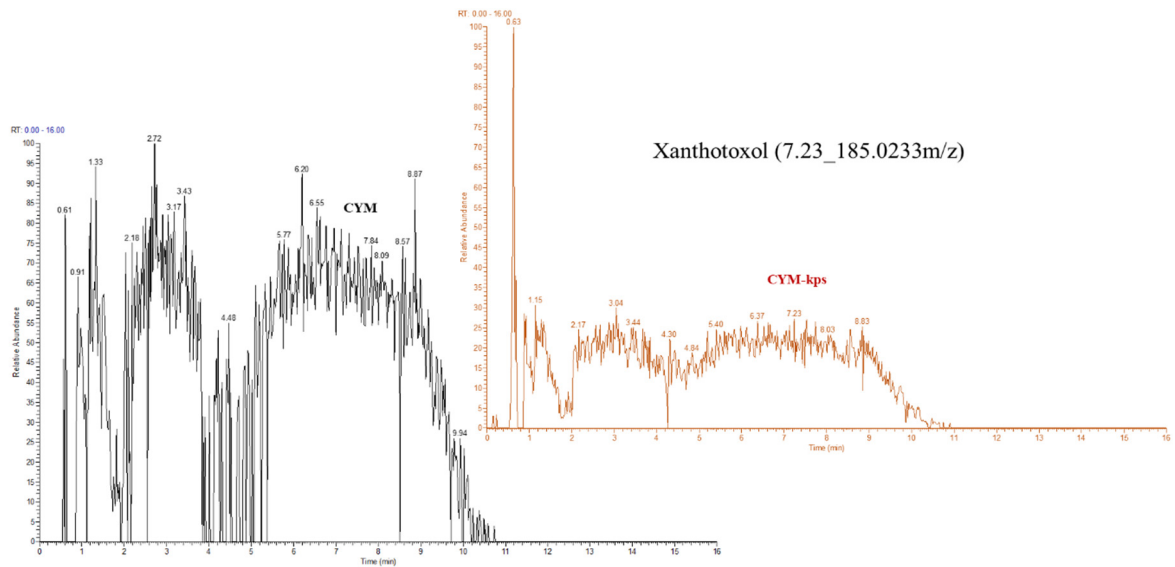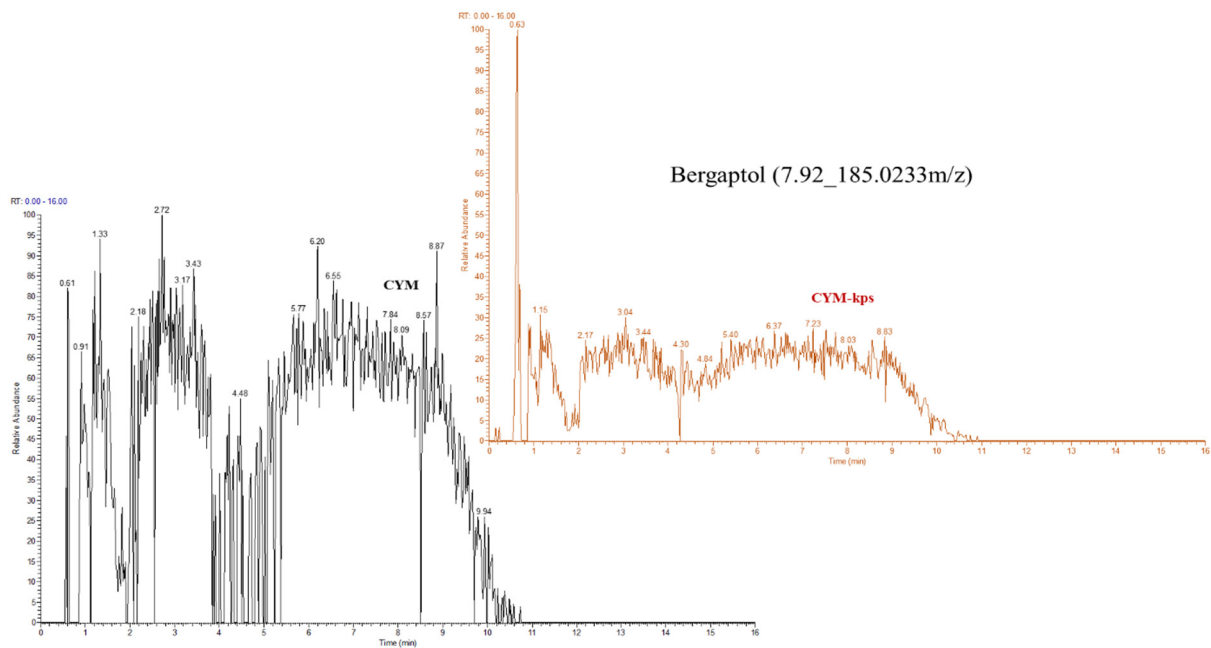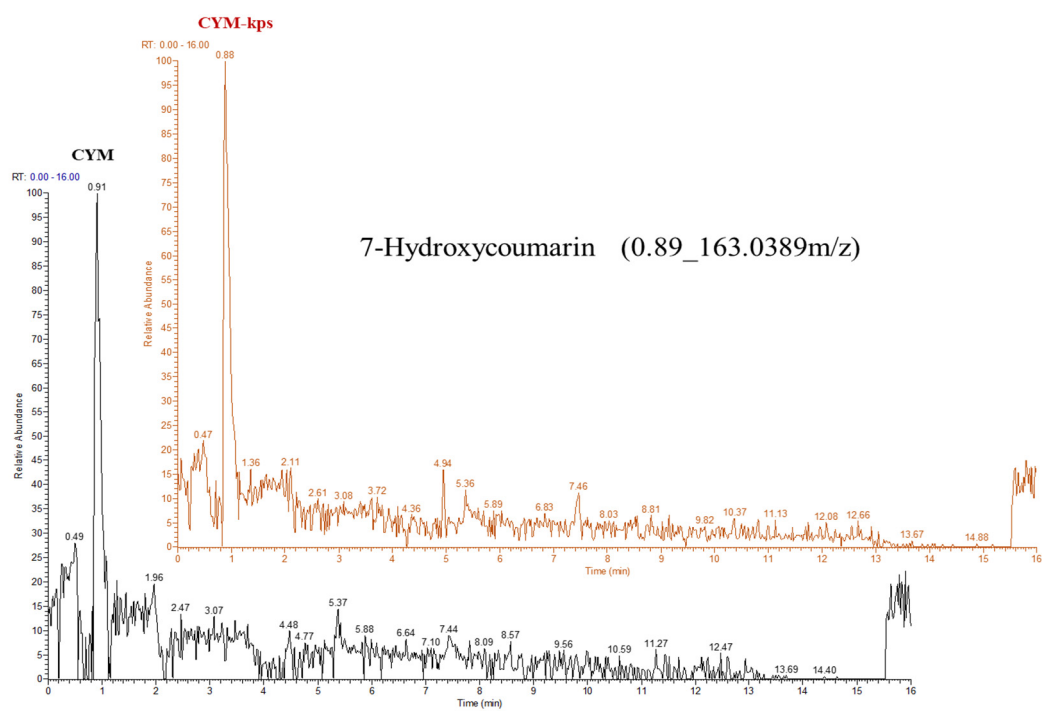

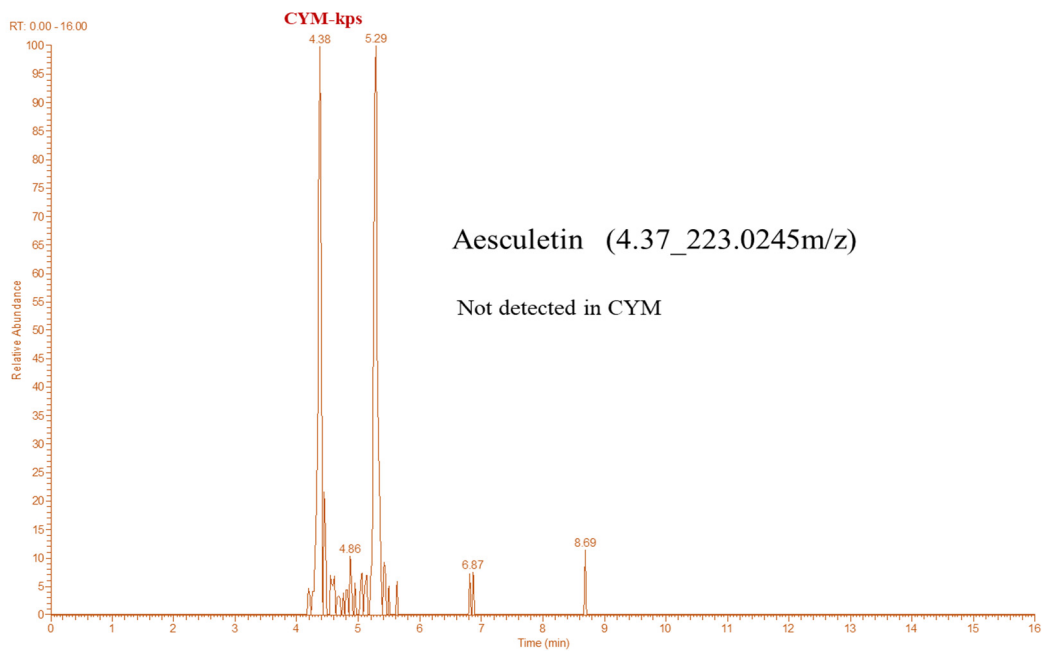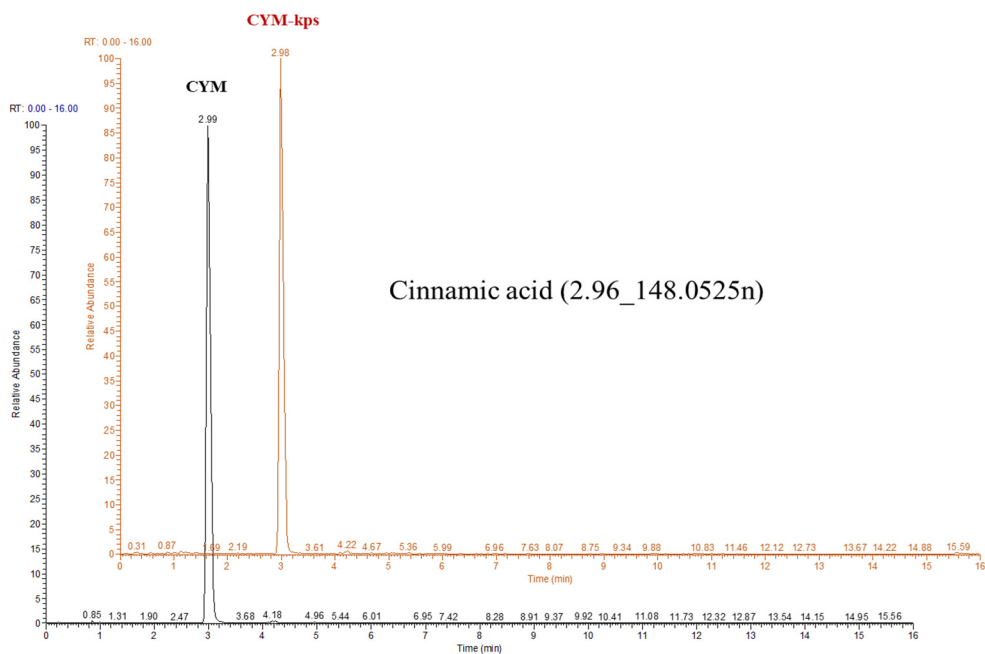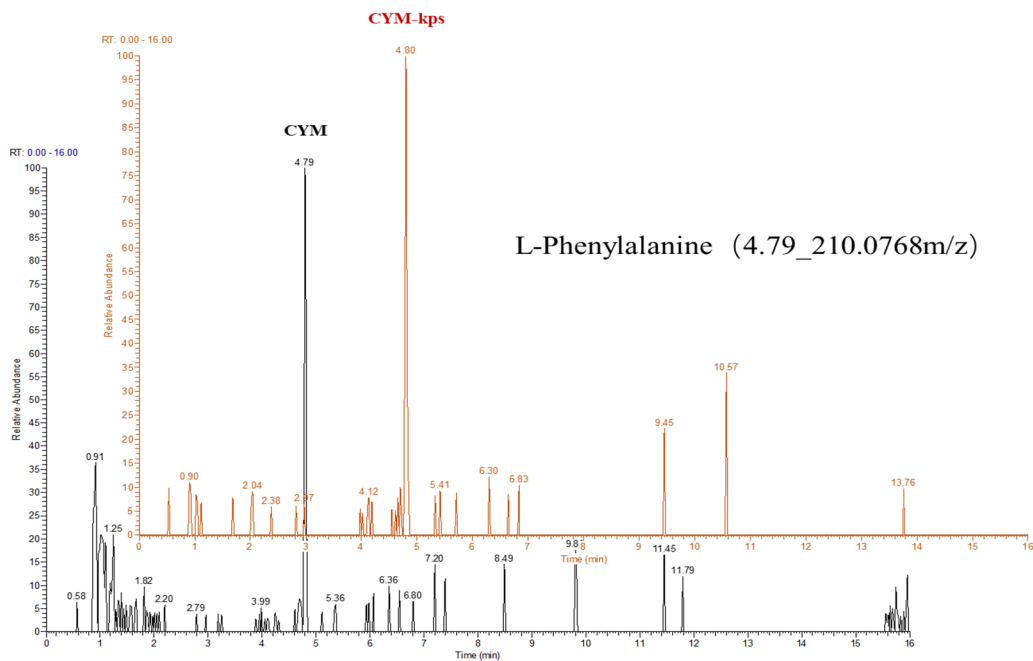

Supplement: Supplementary file 1 [file molecules-27-02334-s001.zip › Supplementary material S2.pdf]
